# Diabetic Retinopathy and Estimated Cerebrospinal Fluid Pressure. The Beijing Eye Study 2011

**DOI:** 10.1371/journal.pone.0096273

**Published:** 2014-05-01

**Authors:** Jost B. Jonas, Ningli Wang, Jie Xu, Ya Xing Wang, Qi Sheng You, Diya Yang, Xiao Bin Xie, Liang Xu

**Affiliations:** 1 Beijing Institute of Ophthalmology, Beijing Tongren Eye Center, Beijing Tongren Hospital, Capital Medical University, Beijing Ophthalmology and Visual Science Key Lab, Beijing, China; 2 Department of Ophthalmology, Medical Faculty Mannheim of the Ruprecht-Karls-University of Heidelberg, Mannheim, Germany; 3 Beijing Tongren Eye Center, Beijing Tongren Hospital, Capital Medical University, Beijing Ophthalmology and Visual Sciences Key Laboratory, Beijing, China; Zhongshan Ophthalmic Center, China

## Abstract

**Purpose:**

The cerebrospinal fluid pressure (CSFP) is a major determinant of central retinal vein pressure and thus of retinal capillary pressure. We tested the hypothesis whether prevalence and severity of diabetic retinopathy are associated with CSFP.

**Methods:**

The population-based Beijing Eye Study 2011 included 3468 individuals with a mean age of 64.6±9.8 years. A detailed ophthalmic examination was performed including fundus photography for the assessment of diabetic retinopathy according. Based on a previous study with lumbar cerebrospinal fluid pressure (CSFP) measurements, CSFP was calculated as CSFP[mmHg] = 0.44xBody Mass Index[kg/m^2^]+0.16 Diastolic Blood Pressure[mmHg]–0.18xAge[Years]−1.91.

**Results:**

In binary regression analysis, presence of diabetic retinopathy was significantly associated with higher levels of HbA1c (*P*<0.001; regression coefficient B:0.25; odds ratio (OR):1.28; 95% confidence interval (CI):1.15,1.43), higher blood concentration of glucose (*P*<0.001; B:0.40;OR:1.49;95%CI:1.36,1.63), longer known duration of diabetes mellitus (*P*<0.001; B:0.14;OR:1.15; 95%CI:1.11,1.19), higher systolic blood pressure (*P*<0.001; B:0.03;OR:1.03;95%CI:1.02,1.04), lower diastolic blood pressure (*P*<0.001; B:−0.06;OR:0.94;95%CI:0.91,0.97), and higher CSFP (*P* = 0.002; B:0.13;OR:1.14;95%CI:1.05,1.24). Severity of diabetic retinopathy was significantly associated with higher HbA1c value (*P*<0.001; standardized coefficient beta: 0.19; correlation coefficient B: 0.07;95%CI:0.05,0.08), higher blood concentration of glucose (*P*<0.001; beta:0.18;B:0.04;95%CI:0.04,0.05), longer known duration of diabetes mellitus (*P*<0.001; beta:0.20;B:0.03;95%CI:0.02,0.03), lower level of education (*P* = 0.001; beta:−0.05;B:−0.02;95%CI:−0.03,−0.01), lower diastolic blood pressure (*P* = 0.002; beta:−0.08;B:−0.001;95%CI:−0.004,−0.001), higher systolic blood pressure (*P* = 0.006; beta:0.06;B:0.001;95%CI:0.000,0.001), and higher CSFP (*P* = 0.006; beta:0.06;B:0.006;95%CI:0.002,0.010).

**Conclusions:**

Higher prevalence and severity of diabetic retinopathy were associated with higher estimated CSFP after adjusting for systemic parameters. Higher CSFP through a higher retinal vein pressure may lead to more marked retinal venous congestion and vascular leakage in diabetic retinae.

## Introduction

Features of diabetic retinopathy are retinal microaneurysms, retinal hemorrhages, retinal edema, retinal vein dilatation, and lipid deposition in the deep retinal layers [1], [2]. Since leakage of lipids, erythrocytes and serum through a vessel wall depends on the pressure in the vessel, since the diameter of a vessel also depends on its internal pressure, and since capillary blood pressure is influenced by the pressure in the draining veins, we assumed that an increased retinal vein pressure is a factor associated with presence and severity diabetic retinopathy. The blood pressure in the central retinal vein inside of the eye is at least as high as the orbital cerebrospinal fluid pressure (CSFP), since the central retinal vein passes, after leaving the eye, through the optic nerve and the orbital cerebrospinal fluid space [3], [4]. We therefore put forward the hypothesis that an increased CSFP (through an elevated retinal vein pressure) is associated with diabetic retinopathy. This hypothesis would fit with the role of arterial hypertension as risk factor for diabetic retinopathy, since a higher arterial blood pressure is associated with a higher CSFP [5], [6]. To test the hypothesis, we conducted a study in which we estimated the CSFP and compared eyes with diabetic retinopathy and eyes without diabetic retinopathy. In previous studies, the correlation between CSFP and age, arterial blood pressure and body mass index was described and a formula allowed estimating the CSFP [6]. We used this formula to assess the CSFP in the participants of our study. We chose a population-based study design to avoid a referral induced bias in the selection of study participants.

## Methods

### Ethics Statement

The Medical Ethics Committee of the Beijing Tongren Hospital approved the study protocol and all participants gave informed written consent.

The Beijing Eye Study 2011 is a population-based cross-sectional study in Northern China [7], [8]. It was carried out in 5 communities in an urban district in the North of Central Beijing and in 3 communities in a rural region south of Beijing. Out of an eligible population of 4403 individuals fulfilling the only inclusion criterion of an age of 50+ years, 3468 (78.8%) individuals (1963 (56.6%) women) participated in the eye examination. The study was divided into a rural part (1633 (47.1%) subjects; 943 (57.7%) women) and an urban part (1835 (52.9%) subjects; 1020 (55.6%) women). The mean age was 64.6±9.8 years (median, 64 years; range, 50–93 years).

For all study participants, an interview was performed with standardized questions on the level of education, quality of life, known major systemic diseases, and other parameters. Fasting blood samples were taken for measurement of blood lipids, glucose and glycosylated hemoglobin HbA1c. Body height and weight and the circumference of the waist and hip were measured. The blood pressure was measured with the participant sitting for at least 5 minutes. The study participants had refrained from smoking and drinking of coffee, tea, or alcohol for at least 3 hours. In addition, any exercise was not performed for the last 30 minutes prior to the blood pressure measurements. A standardized mercury sphygmomanometer was used, and the cuff size was chosen according to the measured circumference of the upper arm [9]. The ophthalmological examinations included measurement of visual acuity, tonometry, slit lamp assisted biomicroscopy of the anterior segment of the eye, biometry applying optical low-coherence reflectometry (Lensstar 900 Optical Biometer, Haag-Streit, 3098 Koeniz, Switzerland), and digital photography of the cornea, lens, fundus and optic nerve head.

Using the fundus photographs (non-stereoscopic 45° photograph of the central fundus and of the optic disc; fundus camera type CR6-45NM, Canon Inc., Ōsta, Tokyo, Japan), diabetic retinopathy was assessed in a masked manner. The diagnosis for each individual was based on the grading of the worse eye per subject. The grading was performed according to the Early Treatment of Diabetic Retinopathy Study (ETDRS) criteria [10]. The minimum criterion for diagnosis of diabetic retinopathy was the presence of at least one microaneurysm. The severity of diabetic retinopathy was graded into mild non-proliferative diabetic retinopathy (20≤ ETDRS level <43 with at least one microaneurysm), moderate non-proliferative diabetic retinopathy (43≤ ETDRS level <53), severe non-proliferative diabetic retinopathy (53≤ ETDRS level <61), and proliferative diabetic retinopathy (ETDRS level ≥61). The photographs were assessed by an experienced and trained ophthalmologist (JX). In case of doubt, the photographs were re-assessed by a panel including several ophthalmologists (JX, LX, YXW, QSY, JBJ). For study purposes, we diagnosed diabetes mellitus as fasting blood glucose concentration ≥7.0 mmol/L, an HbA1c value ≥6%, by a self-reported history of physician diagnosis of diabetes mellitus, or by a history of drug treatment for diabetes (insulin or oral hypoglycemic agents). All subjects diagnosed with diabetic retinopathy had diabetes mellitus.

For the calculation of a formula to estimate the CSFP, we used the lumbar CSFP measurements obtained in a previous pilot study [6]. This was a prospective observational comparative study on patients who consecutively underwent lumbar puncture for diagnosis and treatment of neurological diseases. The lumbar CSFP was measured in a standardized manner at 14∶00 hours. The study included 74 patients with a mean age of 42.0±13.4 years. The mean CSFP was 12.6±4.8 mm Hg (median: 12 mmHg; range: 4–26 mm Hg). The final diagnosis of the patients included diseases such as peripheral neuropathy, multiple sclerosis, unilateral ischemic optic neuropathy and unilateral optic neuritis, in which it was unlikely that the neurological disease was associated with an abnormal CSFP. Out of the total group, we randomly formed a training group consisting of 32 patients, and a testing group including the remaining 42 patients. Due to randomization, the training group and testing group did not differ significantly in age, gender, body height and weight, body mass index, intraocular pressure, retinal nerve fiber layer thickness, and arterial blood pressure (all *P*>0.10). Performing a multivariate analysis in the training group with the lumbar CSFP measurements as dependent variable and age, body mass index and blood pressure as independent variables revealed, that CSFP was best described by the formula of CSFP [mmHg] = 0.44 × Body Mass Index [kg/m^2^] +0.16 × Diastolic Blood Pressure [mmHg] –0.18 × Age [Years]−1.91. The association between higher CSFP and younger age, higher body mass index and higher blood pressure had also been found in other previous investigations [11], [12]. We then tested the formula in the testing group. In this testing group, the measured lumbar CSFP (12.6±4.8 mm Hg) did not differ significantly (*P* = 0.29) from the calculated CSFP (13.3±3.2 mm Hg). The Durbin-Watson value was 2.08. Durbin-Watson values falling into the acceptable range of 1.5 to 2.5 indicate a non-significant autocorrelation for the residuals in the multiple regression models. The intra-class correlation coefficient was 0.71. The Bland-Altman analysis revealed that 40 out of 42 measurements were within the 95% limits of agreement.

Inclusion criteria for the present study were the availability of data on the estimated CSFP and diabetic retinopathy. Statistical analysis was performed using a commercially available statistical software package (SPSS for Windows, version 21.0, IBM-SPSS, Chicago, IL). In a first step, we examined the mean values (presented as mean ± standard deviation). Frequencies were presented as mean ± standard error. In a second step, we performed a univariate analysis of associations between the prevalence of diabetic retinopathy and systemic parameters and ocular parameters. In a third step, we carried out a binary regression analysis, with the presence of diabetic retinopathy as dependent parameter and all those parameters as independent variables which were significantly associated with diabetic retinopathy in univariate analysis. We then dropped in a step-wise manner all those variables from the list of independent parameters, which were no longer significantly associated with the presence of diabetic retinopathy, starting with the parameters with the highest *P*-values. In a fourth step of the analysis, we performed a multivariable analysis of the associations between the severity of diabetic retinopathy. We presented 95% confidence intervals (CI). All *P*-values were 2-sided and were considered statistically significant when the values were less than 0.05.

## Results

Out of the 3468 subjects (6936 eyes) included into the study, data of CSFP and diabetic retinopathy were available for 3355 (96.7%) subjects (6705 (96.7%) eyes) with a mean age of 64.4±9.7 years (median: 63 years; range: 50–93 years), a mean refractive error of −0.22±2.11 diopters (median: 0.25 diopters; range: −22.0 to +7.00 diopters) and a mean axial length of 23.3±1.1 mm (median: 23.1 mm; range: 18.96–30.88 mm). The subjects participating in the study as compared to the non-participating subjects (n = 113) (age: 70.0±11.1 years; refractive error: −1.43±4.05 diopters; axial length: 23.4±2.1 mm) were significantly (*P*<0.001) younger and did not differ significantly in refractive error (*P* = 0.10) nor axial length (*P* = 0.81).

Diabetic retinopathy was detected on the fundus photographs of 163 eyes (2.42±0.19% (95%CI: 2.05, 2.78)) of 98 subjects (2.91±0.29% (95%CI: 2.34, 3.48)). Diabetic retinopathy was of the mild type in 70 (43%) eyes, moderate type in 50 (31%) eyes, severe type in 20 (12%) eyes, and proliferative or status after retinal laser coagulation in 23 (14%) eyes.

In univariate analysis, prevalence of diabetic retinopathy was significantly associated with the systemic parameters of higher body mass index (*P*<0.001), higher levels of glycosylated hemoglobin HbA1c (*P*<0.001), higher blood concentration of glucose (*P*<0.001), higher systolic blood pressure (*P*<0.001), longer known duration of diabetes mellitus (*P*<0.001), urban region of habitation (*P* = 0.001), lower level of education (*P* = 0.003), and higher CSFP (*P* = 0.01); and with the ocular parameters of higher intraocular pressure (*P* = 0.03) and shorter ocular axial length (*P*<0.001) (Table 1). The prevalence of diabetic retinopathy was not significantly associated with age (*P* = 0.69), gender (*P* = 0.85), blood concentrations of high-density lipoproteins (*P* = 0.36), low-density lipoproteins (*P* = 0.73) and triglycerides (*P* = 0.39), ever smoking (*P* = 0.62), and consumption of alcohol (*P* = 0.07).

**Table 1:** Associations between the Presence of Diabetic Retinopathy and Ocular and Systemic Parameters in the Beijing Eye Study.

Parameter	*P*-Value	Odds Ratio	95% Confidence Interval
Body Mass Index (kg/m^2^)	<0.001	1.11	1.07, 1.15
Glycosylated Hemoglobin	<0.001	2.51	2.27, 2.79
Blood Concentration of Glucose (mmol/)	<0.001	1.76	1.65, 1.88
Region of Habitation (Rural/Urban)	<0.001	0.58	0.41, 0.77
Level of Education (1–5)	<0.001	0.75	0.66, 0.85
Known Duration of Diabetes mellitus (Years)	<0.001	1.15	1.13, 1.18
Systolic Blood Pressure (mmHg)	<0.001	1.02	1.01, 1.02
Diastolic Blood Pressure (mmHg)	0.57		
Estimated Cerebrospinal Fluid Pressure (mmHg)	0.01	1.06	1.01, 1.10
Ocular Axial Length (mm)	<0.001	0.64	0.50, 0.81
Intraocular Pressure (mmHg)	0.03	1.06	1.01, 1.12

The binary regression analysis included the presence of diabetic retinopathy as dependent parameter and all those parameters as independent variables which were significantly associated with diabetic retinopathy in the univariate analysis. We then dropped step-by-step those independent parameters which were no longer significantly associated with diabetic retinopathy. After dropping intraocular pressure, presence of glaucoma, level of education, region of habitation, and axial length, presence of diabetic retinopathy remained to be significantly associated with higher levels of HbA1c (*P*<0.001), higher blood concentration of glucose (*P*<0.001), longer known duration of diabetes mellitus (*P*<0.001), higher systolic blood pressure (*P*<0.001), lower diastolic blood pressure (*P*<0.001), and higher CSFP (*P* = 0.002) (Table 2) If body mass index was added to the model, it was not significantly (*P* = 0.93) associated with diabetic retinopathy. In a similar manner, if age was added to the model, it was neither significantly (*P* = 0.93) associated with diabetic retinopathy.

**Table 2:** Results of the Multivariate Analysis of the Association between the Prevalence of Diabetic Retinopathy, Estimated Cerebrospinal Fluid Pressure and Other Systemic Parameters in the Beijing Eye Study.

Parameter	*P*-Value	Odds Ratio	95% Confidence Interval
Estimated Cerebrospinal Fluid Pressure (mmHg)	0.003	1.14	1.05, 1.24
Glycosylated Hemoglobin	<0.001	1.55	1.30,1.85
Blood Concentration of Glucose (mmol/)	<0.001	1.36	1.21, 1.52
Known Duration of Diabetes mellitus (Years)	<0.001	1.15	1.11, 1.18
Systolic Blood Pressure (mmHg)	<0.001	1.03	1.02, 1.04
Diastolic Blood Pressure (mmHg)	<0.001	0.94	0.91, 0.97

**Figure 1:**
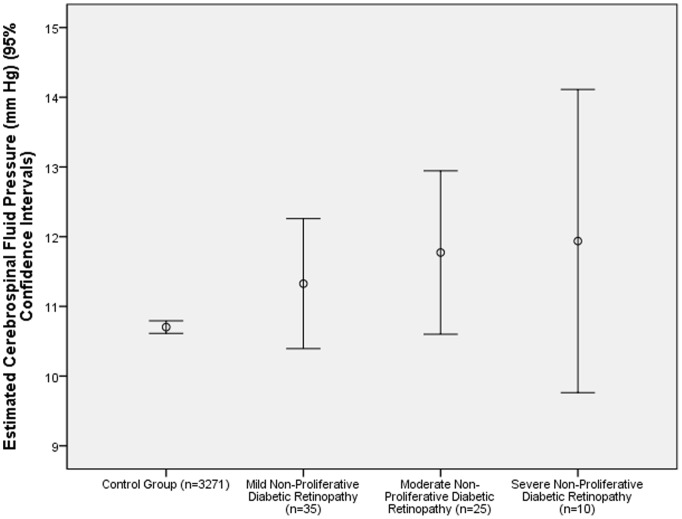
Graph Showing the Distribution of Estimated Cerebrospinal Fluid Pressure and the Severity of Diabetic Retinopathy in the Beijing Eye Study 2011 (Eyes after Retinal Laser Coagulation were Excluded).

The severity of diabetic retinopathy was significantly associated (univariate analysis) with the systemic parameters of higher levels of glycosylated hemoglobin HbA1c (*P*<0.001), higher blood concentration of glucose (*P*<0.001), longer known duration of diabetes mellitus (*P*<0.001), higher systolic blood pressure (*P*<0.001), rural region of habitation (*P* = 0.007), lower level of education (*P* = 0.003), and higher CSFP (*P* = 0.005) (Fig. 1); and with the ocular parameters of higher intraocular pressure (*P* = 0.03), and shorter ocular axial length (*P*<0.001) (Table 3). The severity of diabetic retinopathy was not significantly associated with gender (*P* = 0.86), blood concentrations of cholesterol (*P* = 0.64), high-density lipoproteins (*P* = 0.44), low-density lipoproteins (*P* = 0.71) and triglycerides (*P* = 0.52), ever smoking (*P* = 0.26), and consumption of alcohol (*P* = 0.07), and presence of glaucomatous optic neuropathy (*P* = 0.11).

**Table 3:** Results of the Univariate Analysis of the Association between Stage of Diabetic Retinopathy, Estimated Cerebrospinal Fluid Pressure and Other or Ocular Systemic Parameters in the Beijing Eye Study.

Parameter	*P*-Value	Standardized Coefficient Beta	Regression Coefficient B	95% Confidence Interval
Estimated Cerebrospinal Fluid Pressure (mmHg)	0.05	0.03	0.002	0.001, 0.004
Glycosylated Hemoglobin	<0.001	0.40	0.13	0.12, 0.13
Blood Concentration of Glucose (mmol/)	<0.001	0.37	0.07	0.07, 0.08
Known Duration of Diabetes mellitus (Years)	<0.001	0.19	0.02	0.02, 0.02
Systolic Blood Pressure (mmHg)	<0.001	0.05	0.001	0.00, 0.001
Diastolic Blood Pressure (mmHg)	0.33			
Region of Habitation (Rural/Urban)	<0.001	−0.06	−0.03	−0.04, −0.02
Level of education (1−5)	<0.001	−0.07	−0.02	−0.02, −0.01
Intraocular Pressure (mmHg)	0.004	0.04	0.003	0.001, 0.005
Ocular Axial Length (mm)	<0.001	−0.07	−0.02	−0.03, −0.01

In multivariate analysis, after dropping of the parameters of region of habitation, intraocular pressure and axial length, the severity of diabetic retinopathy remained to be significantly associated with higher HbA1c value (*P*<0.001), higher blood concentration of glucose (*P*<0.001), longer known duration of diabetes mellitus (*P*<0.001), lower level of education (*P* = 0.001), lower diastolic blood pressure (*P* = 0.002), higher systolic blood pressure (*P* = 0.006), and higher CSFP (*P* = 0.006) (Table 4).

**Table 4:** Results of the Multivariate Analysis of the Association between the Stage of Diabetic Retinopathy, Estimated Cerebrospinal Fluid Pressure and Other Systemic Parameters in the Beijing Eye Study.

Parameter	*P*-Value	Standardized Coefficient Beta	Regression Coefficient B	95% Confidence Interval
Estimated Cerebrospinal Fluid Pressure (mmHg)	0.006	0.06	0.006	0.002, 0.010
Glycosylated Hemoglobin	<0.001	0.19	0.07	0.05, 0.08
Blood Concentration of Glucose (mmol/)	<0.001	0.18	0.04	0.04, 0.05
Known Duration of Diabetes mellitus (Years)	<0.001	0.20	0.03	0.02, 0.03
Level of Education (1−5)	0.001	−0.05	−0.02	0.03, −0.01
Systolic Blood Pressure (mmHg)	0.006	0.06	0.001	0.000, 0.001
Diastolic Blood Pressure (mmHg)	0.002	−0.08	−0.001	−0.004, −0.001

## Discussion

In our population-based study, presence of diabetic retinopathy was significantly (*P* = 0.002) associated with higher CSFP after adjusting for higher levels of HbA1c, higher blood concentration of glucose, longer known duration of diabetes mellitus, higher systolic blood pressure and lower diastolic blood pressure. Correspondingly, the severity of diabetic retinopathy was significantly associated with higher CSFP (*P* = 0.006) after adjusting for higher levels of HbA1c, higher blood concentration of glucose, longer known duration of diabetes mellitus, lower level of education, higher systolic blood pressure and lower diastolic blood pressure.

The findings of our study agree with a multitude of previous investigations that increased blood pressure, elevated HbA1c levels and a long duration of diabetes mellitus were major risk factors for the presence and severity of diabetic retinopathy [13]–[16]. Our study additionally suggested that higher CSFP was an additional factor associated with the presence and severity of diabetic retinopathy in our population-based study participants.

The blood pressure in the central retinal vein inside of the eye is at least as high as the CSFP, since the central retinal vein, after leaving the eye, passes through the optic nerve and the orbital cerebrospinal fluid space. An elevated CSFP is therefore associated with an elevated intraocular retinal vein pressure as shown in previous ophthalmodynamometric studies [3], [4]. The elevated retinal vein pressure in patients with higher CSFP will be associated with a higher retinal capillary blood pressure potentially explaining the increased prevalence of retinal hemorrhages, edema and lipid exudates as part of diabetic retinopathy in patients with diabetes mellitus in our study population.

Besides diabetic retinopathy, retinal vein occlusions are another hemorrhagic retinopathy and are characterized by retinal hemorrhages, retinal edema, lipid exudation and venous dilatation. Previous ophthalmodynamometric studies have revealed a markedly elevated retinal vein pressure in eyes with retinal vein occlusions [17]. Recently, an elevated CSFP was found to be associated with the incidence of retinal vein occlusions (own data). This finding fits with the observation in the present study. The association of retinal vein occlusions with higher CSFP could explain why arterial blood pressure is indirectly associated with the prevalence of retinal vein occlusions, since higher arterial blood pressure is correlated with higher CSFP. In the case of diabetic retinopathy it has remained unclear, whether elevated blood pressure directly leads to diabetic retinopathy or - at least partially - indirectly through an increased CSFP.

The results of our study agree with clinical observations and studies that increased brain pressure can be associated with retinal hemorrhages. In the case of an acute subarachnoidal hemorrhage with a marked increase in intracranial pressure, retinal hemorrhages and an intravitreal bleeding can develop as part of Terson’s syndrome [18], [19]. In other neurological disorders associated with brain edema and increased brain pressure such as mountain sickness, retinal hemorrhages can typically occur [20]. In arterial hypertensive retinopathy with retinal hemorrhages in stage III and papilledema in stage IV, the markedly elevated blood pressure may induce a pronounced increase in intracranial pressure which can explain the occurrence of retinal hemorrhages and papilledema. In a parallel manner, a recent study showed that the retinal vein diameter and the ratio of retinal vein-to-artery diameter depended on the estimated CSFP [21].

The findings of our study suggested that an altered CSFP could lead to an increased prevalence and severity of diabetic retinopathy with retinal hemorrhages and macular edema. While retinal capillary pressure is likely to play a role, previous studies have also suggested a role for ocular perfusion pressure which incorporates intraocular pressure. In a previous investigation, Quigely and Cohen combined the Ohm, Poiseuille, and Murray laws and found that the myopic arteriolar tree would produce a 16% greater pressure attenuation than that of emmetropic controls with a linear relationship between mean pressure attenuation index and axial length [22]. Since myopia has been shown to be protective against diabetic retinopathy, Quigley and Cohen postulated that the low-end arteriolar pressure in myopic eyes could be protective mechanism against diabetic retinopathy in myopia [23]. In contrast, Stodtmeister and colleagues recently estimated that an increased retinal vein pressure reduces the ocular perfusion pressure [24]. If the ocular perfusion pressure is decreased, the risk for ischemic retinopathies such as diabetic retinopathy may increase. A recent study suggested that increased CSFP is associated with an increased diameter of the retinal veins what is a surrogate for an increased retinal vein pressure [21]. Persons with higher CSFP may thus have a lower ocular perfusion pressure and potentially due to that mechanism, a higher prevalence of diabetic retinopathy.

If the association between higher CSFP and presence and severity of diabetic retinopathy is further clarified in future studies, one may address the question whether lowering of CSFP by drugs such as systemic carbonic anhydrase inhibitors may have a therapeutically positive effect on diabetic retinopathy.

Potential limitations of our study should be mentioned. First, the whole study depended on the estimation of CSFP being derived from a multivariate formula incorporating body mass index, diastolic blood pressure, and age. The result of this formula was then termed CSFP and it correlated with the presence and severity of diabetic retinopathy in multivariate analyses. Although the estimated CSFP was primarily just the result of a mathematical equation, the calculated CSFP values correlated well with invasively measured CSFP values in the independent test group of the pilot study. Nonetheless, the unknown general validity of the equation to estimate the CSFP may be the most important limiting factor of our study. Second, some of the components of the formula to estimate the CSFP were by themselves associated with diabetic retinopathy. It has therefore remained unclear whether diabetic retinopathy was correlated with CSFP or with the individual components that were used to calculate CSFP. In the multivariate analysis, the presence of diabetic retinopathy was significantly associated with higher CSFP after adjusting for systolic and diastolic blood pressure, while diabetic retinopathy was not associated with age or body mass index in the model. Since the three parameters of age, blood pressure and body mass index influence CSFP and since body mass index and blood pressure were also associated with diabetic retinopathy in univariate analysis, it was not possible to distinguish between a direct effect of CSFP on the prevalence of diabetic retinopathy and a secondary effect by the underlying basic parameters. Such a question may be addressed in a study comparing patients who have diabetic retinopathy and who have elevated or normal CSFP as measured by direct lumbar puncture. Such a design is however not possible to implement in a population-based investigation. Third, as for any population-based study, the rate of non-participation or non-availability of examination results can matter. In our study, the participation rate was 78.8% what may be acceptable. Fourth, blood pressure, although measured under standardized conditions, was determined only once, so that the question arises how representative this single measurement was for the subject’s blood pressure in general.

In conclusion, higher prevalence and severity of diabetic retinopathy were associated with higher estimated CSFP after adjusting for systemic parameters. Higher CSFP through a higher central retinal vein pressure may lead to more marked retinal venous congestion and vascular leakage in diabetic retinae. Future studies may address whether lowering of a slightly elevated CSFP may be helpful for the treatment of diabetic retinopathy.
